# Determination of Abraham model solute descriptors for the monomeric and dimeric forms of trans-cinnamic acid using measured solubilities from the Open Notebook Science Challenge

**DOI:** 10.1186/s13065-015-0080-9

**Published:** 2015-03-22

**Authors:** Jean-Claude Bradley, Michael H Abraham, William E Acree, Andrew SID Lang, Samantha N Beck, David A Bulger, Elizabeth A Clark, Lacey N Condron, Stephanie T Costa, Evan M Curtin, Sozit B Kurtu, Mark I Mangir, Matthew J McBride

**Affiliations:** Department of Chemistry, Drexel University, Disque Hall Rm. 305. 3141 Chestnut Street, Philadelphia, PA 19104 USA; Department of Chemistry, University College London, 20 Gordon Street, London, WC1H OAJ UK; Department of Chemistry, University of North Texas, 1155 Union Circle Drive #305070, Denton, TX 76203 USA; Department of Computing and Mathematics, Oral Roberts University, Tulsa, OK 74171 USA

## Abstract

**Background:**

Calculating Abraham descriptors from solubility values requires that the solute have the same form when dissolved in all solvents. However, carboxylic acids can form dimers when dissolved in non-polar solvents. For such compounds Abraham descriptors can be calculated for both the monomeric and dimeric forms by treating the polar and non-polar systems separately. We illustrate the method of how this can be done by calculating the Abraham descriptors for both the monomeric and dimeric forms of trans-cinnamic acid, the first time that descriptors for a carboxylic acid dimer have been obtained.

**Results:**

Abraham descriptors were calculated for the monomeric form of trans-cinnamic acid using experimental solubility measurements in polar solvents from the Open Notebook Science Challenge together with a number of water-solvent partition coefficients from the literature. Similarly, experimental solubility measurements in non-polar solvents were used to determine Abraham descriptors for the trans-cinnamic acid dimer.

**Conclusion:**

Abraham descriptors were calculated for both the monomeric and dimeric forms of trans-cinnamic acid. This allows for the prediction of further solubilities of trans-cinnamic acid in both polar and non-polar solvents with an error of about 0.10 log units.

**Graphical abstract:**
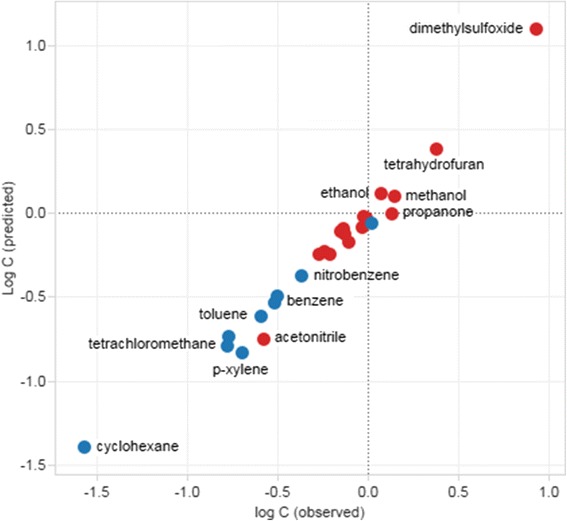
Molar concentration of trans-cinnamic acid in various polar and non-polar solvents.

## Background

The Abraham solvation parameter model describes solute transfer between two condensed phases, or between a condensed phase and a gas phase. Specific chemical and biological processes that have been described by the basic model include water-to-organic solvent and gas-to-organic solvent partition coefficients [1], blood-to-body tissue/fluid and gas-to-body tissue/fluid partition coefficients [2], skin permeability coefficients [3], median lethal concentrations of organic compounds for toxicity towards specific aquatic organisms [3], nasal pungency thresholds [3], Draize eye irritation scores [3], and the minimum alveolar concentration for inhalation anthesia towards rats [3]. Expressed in terms of partition coefficients the Abraham general solvation equations can be formulated as:

1 $$ \log\ {\mathrm{P}}_{\mathrm{s}}=\mathrm{c}+\mathrm{e}\ \mathrm{E}+\mathrm{s}\ \mathrm{S}+\mathrm{a}\ \mathrm{A}+\mathrm{b}\ \mathrm{B}+\mathrm{v}\ \mathrm{V}, $$

2 $$ \log\ {\mathrm{K}}_{\mathrm{s}}=\mathrm{c}+\mathrm{e}\ \mathrm{E}+\mathrm{s}\ \mathrm{S}+\mathrm{a}\ \mathrm{A}+\mathrm{b}\ \mathrm{B}+\mathrm{l}\ \mathrm{L}, $$

where P_s_ is a water-solvent partition coefficient of a solute, K_s_ is a gas-solvent partition coefficient, E, S, A, B, V, and L are the solute descriptors and c, e, s, a, b, v and l are coefficients that describe the particular water-solvent or gas-solvent process. The solute descriptors each describe an important solute property: E represents the excess molar refractivity in units of (cm^3^ per mol)/10, S represents the dipolarity/polarity of the solute, A and B represent the hydrogen bond acidity and basicity respectively, V is the solute’s McGowan characteristic volume in units of (cm^3^ per mol)/100 and L is the logarithm of the gas-hexadecane partition coefficient at 298 K. [4,5]

The solute descriptor V is the easiest to obtain as it can be calculated directly from structure. It is equal to the McGowan characteristic volume (cm^3^ per mol)/100 [6]. V encodes sized-related solvent-solute dispersion interactions, including a measure of the solvent cavity term that will accommodate the dissolved solute.

The solute descriptor E, the excess molar refractivity, can be calculated from a refractive index at 293 K for a compound that is liquid at 293 K [4]. For other solutes E can be predicted, either directly using Absolv, part of ACD Labs proprietary ACD/ADME Suite [7], or through the predicted molar refractivity, freely available for individual compounds through ChemSpider [8], or some other source, such as the Open Source Chemistry Development Kit [9]. Another useful method for estimating E is through summation of structural fragments from compounds with known values of E.

Equation (1) can be applied to saturated molar concentrations, C_s_, of a compound in various organic solvents through Equation (3),

3 $$ {\mathrm{P}}_{\mathrm{s}}={\mathrm{C}}_{\mathrm{s}}/{\mathrm{C}}_{\mathrm{w}} $$

where C_w_ is the aqueous solubility of the compound. If the aqueous solubility is unavailable it can either be left unknown and determined by regression or predicted using ACD Labs ACD/ADME Suite or through the freely available VCC Labs ALOGPS webservice [10].

The solute descriptors S, A, and B can also be predicted [7,11-13] or in limited cases determined experimentally [14,15]. However, accurate results, in general much more accurate than predicted values, are easily obtained by using regression with measured solubilities and/or partition coefficient values [1].

Finally, we note that the applicability of the Abraham model to the solubility of crystalline organic solutes assumes three conditions. Firstly, the solute has the same form when dissolved in any solvent, including water. That is, we assume no solvate, hydrate, or complex formation. Secondly, the secondary medium coefficient must be at or near unity. This condition generally restricts the model to solutes that are not too soluble. Thirdly, if the solute ionizes in water, the aqueous solubility, C_w_, is taken to be that of the neutral form. The second restriction may not be as important as initially believed. The Abraham solvation parameter model has shown remarkable success in correlating the solubility of several very soluble crystalline solutes. For example, Equations (1) and (2) described the molar solubility of 1,4-dichloro-2-nitrobenzene in 24 organic solvents to within overall standard deviations of 0.128 and 0.119 log units, respectively [16]. Standard deviations for aspirin dissolved in 13 alcohols, 4 ethers, and ethyl ethanoate were 0.123 and 0.138 log units [17]. 1,4-Dichloro-2-nitrobenzene and aspirin exhibited solubilities exceeding 1 molar in several of the organic solvents studied.

The Open Notebook Science Challenge [18] contains a valuable collection of Open Data (CC0 1.0 License: See the creative commons website for more information about this license) solubility data that could be used to determine Abraham descriptors for a large number of compounds. We illustrate the utility of the Open Notebook Science Challenge data by determining the Abraham descriptors for both the monomeric and dimeric forms of trans-cinnamic acid. The current study represents the first time that we have calculated the solute descriptors for carboxylic acid dimers. Solute descriptors are required input parameters in order to predict solute solubilities, partition coefficients, and other chemical/biological properties for which Abraham model correlations have been developed.

## Methods

The measured solubility values presented here are from the Open Notebook Science Challenge [18], an Open Science project to collect and measure the solubility of organic compounds in organic solvents, ran by Jean-Claude Bradley, and sponsored by the Royal Society of Chemistry, Sigma Aldrich, Submeta, and Nature. The method and materials used to determine the solubility values varied by experiment and researcher and can be found in the Open Notebook [19].

In addition to the measured solubility values outlined above, we collected solubility values from the literature [20-24] and partition coefficients from Bio-Loom [25]. All values (mole fraction, mass fraction and mass ratio) were converted to molarity for ease of comparison.

The combined collection numbered 69 trans-cinnamic acid/solvent values (molar concentrations) at temperatures ranging from 19.5 C to 28 C. The solubility values were all converted to values at 25 C using the Buchowski equation with the assumption of miscibility at solute melting point [26]. Multiple measurements for the same solvent were averaged (with a mean deviation of 0.067 M) giving a total of 30 solute/solvent values for trans-cinnamic acid, see Table 1 below.

**Table 1:** **Solubilities of trans-cinnamic acid**

**Solvent**	**Molar concentration**
Water	0.004
Cyclohexane	0.027
Carbon tetrachloride	0.167
Trifluoroethanol	0.168
m-xylene	0.201
Toluene	0.253
Acetonitrile	0.263
Tetrachloroethylene	0.277
Benzene	0.303
Chlorobenzene	0.314
Nitrobenzene	0.429
1-octanol	0.537
Diethyl ether	0.575
Trichloroethylene	0.59
Propyl acetate	0.609
Pentachloroethane	0.617
2-butanol	0.705
1-pentanol	0.725
1-butanol	0.735
Ethyl acetate	0.775
2-pentanol	0.922
1-propanol	0.934
2-propanol	0.967
Chloroform	1.034
Tetrachloroethane	1.149
Ethanol	1.167
Acetone	1.337
Methanol	1.384
THF	2.367
DMSO	8.423

The case of cinnamic acid is interesting as it conflicts with our conditions of applicability, above. As with carboxylic acids in general, cinnamic acid dimerizes in the less polar solvents, especially in the less polar aprotic solvents. Experimental dimerization constants, K_dimer_, based on Equation (4) often differ somewhat for the same compound in the same solvent, but whatever the actual value it is evident that at the saturated solubility concentrations, benzoic acid, and by analogy cinnamic acid, will be dimerized in non-polar aprotic solvents. For example K_dimer_ for benzoic acid in cyclohexane is 11300, in tetrachloromethane is 5010 and in benzene is 590 [27].

4 $$ 2\ \mathrm{H}\mathrm{A}={\left(\mathrm{H}\mathrm{A}\right)}_2 $$

We can use this difficulty to advantage by choosing polar solvents for the determination of descriptors for cinnamic acid monomer and by choosing non-polar solvents for the determination of descriptors for cinnamic acid dimer. A few solvents were excluded altogether as they currently do not have Abraham solvent parameters: pentachloroethane, tetrachloroethane, tetrachloroethylene, and trichloroethylene.

### Calculating the Abraham descriptors for cinnamic acid monomer

As input we used solubility data in Table 1 for the polar solvents where cinnamic acid is expected to exist largely in monomeric form, together with a number of direct partition coefficients [25]. Although the latter are partitions from water to non-polar solvents, they still refer to cinnamic acid monomer because the experimental determination has either been carried out at low solute concentration or has been extrapolated to low solute concentration. The direct log P_s_ values that we use [25] are in Table 2.

**Table 2:** **Values of water-solvent partition coefficients, as log P**_**s**_ **, for trans-cinnamic acid monomer**

**Solvent**	**Log P**_**s**_
Octan-1-ol, wet	2.13
Trichloromethane	1.20
Tetrachloromethane	0.40
Cyclohexane	−0.25
Diethyl ether, wet	1.92

The value for E was determined from structure, by comparing cinnamic acid fragment-wise with compounds that have known values for E; ethyl benzoate (E = 0.689), ethyl cinnamate (E = 1.102), and benzoic acid (E = 0.730). The E solute descriptors for ethyl benzoate and benzoic acid differ by 0.041, with benzoic acid having the larger E value. Maintaining the same difference between the E solute descriptors for ethyl cinnamate and trans-cinnamic acid then gives E = 1.14 for trans-cinnamic acid (rounded to the hundredths place) [11]. The solute volume descriptor, calculated from the McGowan characteristic volume, is given by V = 1.1705. We can transform all the P_s_ values into values of the gas-solvent partition coefficient K_s_ through Equation (5), where K_w_ is the dimensionless gas-water partition coefficient

5 $$ {\mathrm{P}}_{\mathrm{s}}={\mathrm{K}}_{\mathrm{s}}/{\mathrm{K}}_{\mathrm{w}} $$

We then have a total of 21 values of log P_s_, 5 being the number of partition coefficient measurements and 16 being the number of values derived from solubility ratios, using Equation (5), with log C_w_ taken as −2.40 [18]. These can be converted into 21 values of log K_s_. We also have two equations for log K_w_, one in terms of V (Equation 1) and one in terms of L (Equation 2), and an equation for GLC retention data [28] thus leading to a total of 45 equations. The unknowns are S, A, L and log K_w_. The set of 45 equations were solved by regression to yield the values of the four unknowns that gave the best fit of experimental and calculated properties, exactly as described before [29,30].

### Calculating the Abraham descriptors for cinnamic acid dimer

The input data is now restricted to solubilities in the less polar solvents where cinnamic acid is expected to exist predominantly in dimeric form. We do not know the solubility of cinnamic acid dimer in water, and so log C_w_ is another unknown quantity to be obtained by regression. We have solubilities in nine non-polar solvents, nine corresponding values of log P_s_ and two equations for log K_w_ giving a total of 20 equations. The value of V for cinnamic acid dimer was obtained in the usual way for a compound of molecular formula C_18_H_16_O_4_ as V = 2.2098. There are a number of aromatic liquid carboxylic acids, with known values of the refractive index at 293 K. These values for the pure liquids will refer to the dimeric form of the carboxylic acid, and can be used to calculate E in the usual way [4] for the dimer. The value for E for the dimeric form can also be obtained by addition of fragments, as we have done for cinnamic acid monomer. We find that the two E-values are related through

6 $$ {\mathrm{E}}_{\mathrm{dimer}}=-0.418+1.839\ {\mathrm{E}}_{\mathrm{monomer}} $$

For cinnamic acid, with E_monomer_ = 1.14 the value of E_dimer_ is 1.68. The unknowns are then S, A, B, L, log K_w_ and log C_w_ so that it is easily possible to obtain a solution for the 20 simultaneous equations by regression.

## Results and discussion

The obtained descriptors for cinnamic acid monomer and cinnamic acid dimer are in Table 3, together with values for benzoic acid (monomer) as a comparison. The statistical fits are very good, and the 20 or 45 log P_s_ and log K_s_ values are fitted with a standard deviation (SD) of about 0.1 log units. As expected, the A-descriptor for cinnamic acid dimer (0.24) is much less than that for twice the monomer (1.22) because the two OH protons are internally bonded and are less available for bonding to an external hydrogen bond base. The other descriptors for cinnamic acid dimer are also as expected. A comparison of descriptors for cinnamic acid and benzoic acid monomers shows quite close agreement. The B-descriptor (hydrogen bond basicity) of cinnamic acid is a little more than that of benzoic acid due to the extra C = C group, and this also slightly increases the S-descriptor and the L-descriptor.

**Table 3:** **Descriptors for monomeric and dimeric cinnamic acid, and for monomeric benzoic acid**

**Cinnamic acid**	**E**	**S**	**A**	**B**	**V**	**L**	**Log K**_**w**_	**N**	**SD**
Monomer	1.14	1.12	0.61	0.50	1.1705	5.79	6.14	45	0.100
Dimer	1.68	1.07	0.24	0.94	2.2098	10.30	6.29	20	0.087
Benzoic acid	0.73	0.90	0.59	0.40	0.9317	4.66	5.10		

**Figure 1:**
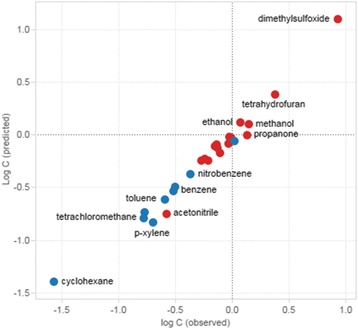
**Observed and fitted solubilities for trans-cinnamic acid.** Red is for dimer in non-polar solvents.

The SD values for the two sets of total equations are quite good but we decided to obtain the statistics for just the solubility data. Details are in Table 4 for the calculations of the cinnamic acid monomer. We include data on the log P_s_ values, but the statistics are exactly the same as for the solubilities. For the 16 solubilities, the average error (AE) between observed and fitted values is 0.006, the absolute average error (AAE) is 0.055 and the SD is 0.078 log units. Thus from the descriptors in Table 3 and the coefficients for the relevant equations, further solubilities of monomeric cinnamic acid in a large numer of polar solvents can be predicted to about 0.10 log units. The corresponding data for the cinnamic acid dimer are in Table 5. For the nine solubilities AE = 0.003, AAE = 0.053 and SD = 0.084 log units, so that solubilities in non-polar solvents can be predicted, again to within about 0.10 log units. It is interesting that the fitted and observed solubility in trifluoroethanol agree to 0.039 log units. An illustration of the results from Tables 4 and 5 can be seen in Figure 1, where the blue circles correspond to non-polar solvents and the red circles correspond to polar solvents.

**Table 4:** **Observed and fitted solubilities for trans-cinnamic acid monomer in polar solvents**

	**Log P**_**s**_		**Log C**_**s**_	
Solvent	Calc	Obs	Calc	Obs
Methanol	2.499	2.541	0.099	0.141
Ethanol	2.515	2.467	0.115	0.067
Propanol	2.381	2.370	−0.019	−0.030
Butanol	2.278	2.266	−0.122	−0.134
Pentanol	2.311	2.260	−0.089	−0.140
Octanol	2.159	2.130	−0.241	−0.270
Propan-2-ol	2.371	2.385	−0.029	−0.015
Butan-2-ol	2.293	2.248	−0.107	−0.152
Pentan-2-ol	2.313	2.365	−0.087	−0.035
Diethylether	2.174	2.160	−0.226	−0.240
Tetrahydrofuran	2.786	2.774	0.386	0.374
Ethyl acetate	2.228	2.289	−0.172	−0.111
Propyl acetate	2.158	2.185	−0.242	−0.215
Propanone	2.400	2.526	0.000	0.126
Acetonitrile	1.653	1.820	−0.747	−0.580
Dimethylsulfoxide	3.497	3.326	1.097	0.925

**Table 5:** **Observed and fitted solubilities for trans-cinnamic acid dimer in non-polar solvents**

	**Log P**_**s**_		**Log C**_**s**_	
Solvent	Calc	Obs	Calc	Obs
Trichloromethane	5.598	5.672	−0.059	0.015
Tetrachloromethane	4.870	4.880	−0.787	−0.777
Cyclohexane	4.264	4.088	−1.393	−1.569
Benzene	5.125	5.138	−0.532	−0.519
Toluene	5.045	5.060	−0.612	−0.597
p-xylene	4.826	4.960	−0.831	−0.697
Chlorobenzene	5.162	5.154	−0.495	−0.503
Nitrobenzene	5.285	5.289	−0.372	−0.368
Trifluoroethanol	4.921	4.882	−0.736	−0.775

Although we refer to solvents that support formation of the dimer as ‘non-polar’ solvents, the main distinguishing factor between solvents that support the dimer and those that support the monomer is the hydrogen bond basicity of the solvent. If the solvent is a hydrogen bond base, it will form solvent-solute hydrogen bonds with the OH group and will break up the dimer into the monomeric form. Trifluoroethanol as a solvent is an extremely weak hydrogen bond base. Marcus [31] gives values of the Kamlet-Taft solvent hydrogen bond basicity, β, as methanol (0.66), diethyl ether (0.47), propanone (0.43) propyl acetate (0.40), acetonitrile (0.40), nitrobenzene (0.30), trichloromethane (0.10), benzene (0.10), cyclohexane (0.00) and trifluoroethanol (0.00). It seems that for saturated solutions of cinnamic acid in solvents with β > 0.35 the monomer is mainly present but when the solvent β < 0.35 the dimer is mainly present.

## Conclusion

We have determined Abraham solute descriptors for trans-cinnamic acid using solubility values measured using Open Notebook Science supplemented with values reported in the literature and with values of partition coefficients from the literature. For compounds that are not dimerized it is quite easy to perform these calculations using just solubility data. We have determined Abraham solute descriptors for the dimer of trans-cinnamic acid using just solubilities from the Open Notebook Science Challenge supplemented with values reported in the literature. This is the first time that descriptors have been assigned to carboxylic acid dimers. The Open Notebook Science Challenge details solubilities for a number of compounds that are easier to work with than cinnamic acid, because they do not form dimers. Those wishing to calculate Abraham solute descriptors for other compounds in a similar fashion can use the solubility data in the Open Notebook Science Challenge database to do so.
